# Rho of Plants patterning: linking mathematical models and molecular diversity

**DOI:** 10.1093/jxb/erad447

**Published:** 2023-11-14

**Authors:** Eva E Deinum, Bas Jacobs

**Affiliations:** Mathematical and Statistical Methods (Biometris), Plant Science Group, Wageningen University, 6708 PB Wageningen, The Netherlands; Mathematical and Statistical Methods (Biometris), Plant Science Group, Wageningen University, 6708 PB Wageningen, The Netherlands; INRAE-Bordeaux, France

**Keywords:** Cluster coexistence, cytoskeleton, lipid nanodomains, mathematical models, pattern formation, Rho of Plants (ROP), type-I/II ROPs

## Abstract

ROPs (Rho of Plants) are plant specific small GTPases involved in many membrane patterning processes and play important roles in the establishment and communication of cell polarity. These small GTPases can produce a wide variety of patterns, ranging from a single cluster in tip-growing root hairs and pollen tubes to an oriented stripe pattern controlling protoxylem cell wall deposition. For an understanding of what controls these various patterns, models are indispensable. Consequently, many modelling studies on small GTPase patterning exist, often focusing on yeast or animal cells. Multiple patterns occurring in plants, however, require the stable co-existence of multiple active ROP clusters, which does not occur with the most common yeast/animal models. The possibility of such patterns critically depends on the precise model formulation. Additionally, different small GTPases are usually treated interchangeably in models, even though plants possess two types of ROPs with distinct molecular properties, one of which is unique to plants. Furthermore, the shape and even the type of ROP patterns may be affected by the cortical cytoskeleton, and cortex composition and anisotropy differ dramatically between plants and animals. Here, we review insights into ROP patterning from modelling efforts across kingdoms, as well as some outstanding questions arising from these models and recent experimental findings.

## Teaser

ROPs are central membrane patterning proteins in plants. We relate insights from and assumptions of ROP models and their yeast/animal counterparts to recent experimental findings and plant-specific molecular diversity.

## Introduction

**Fig. 1.**
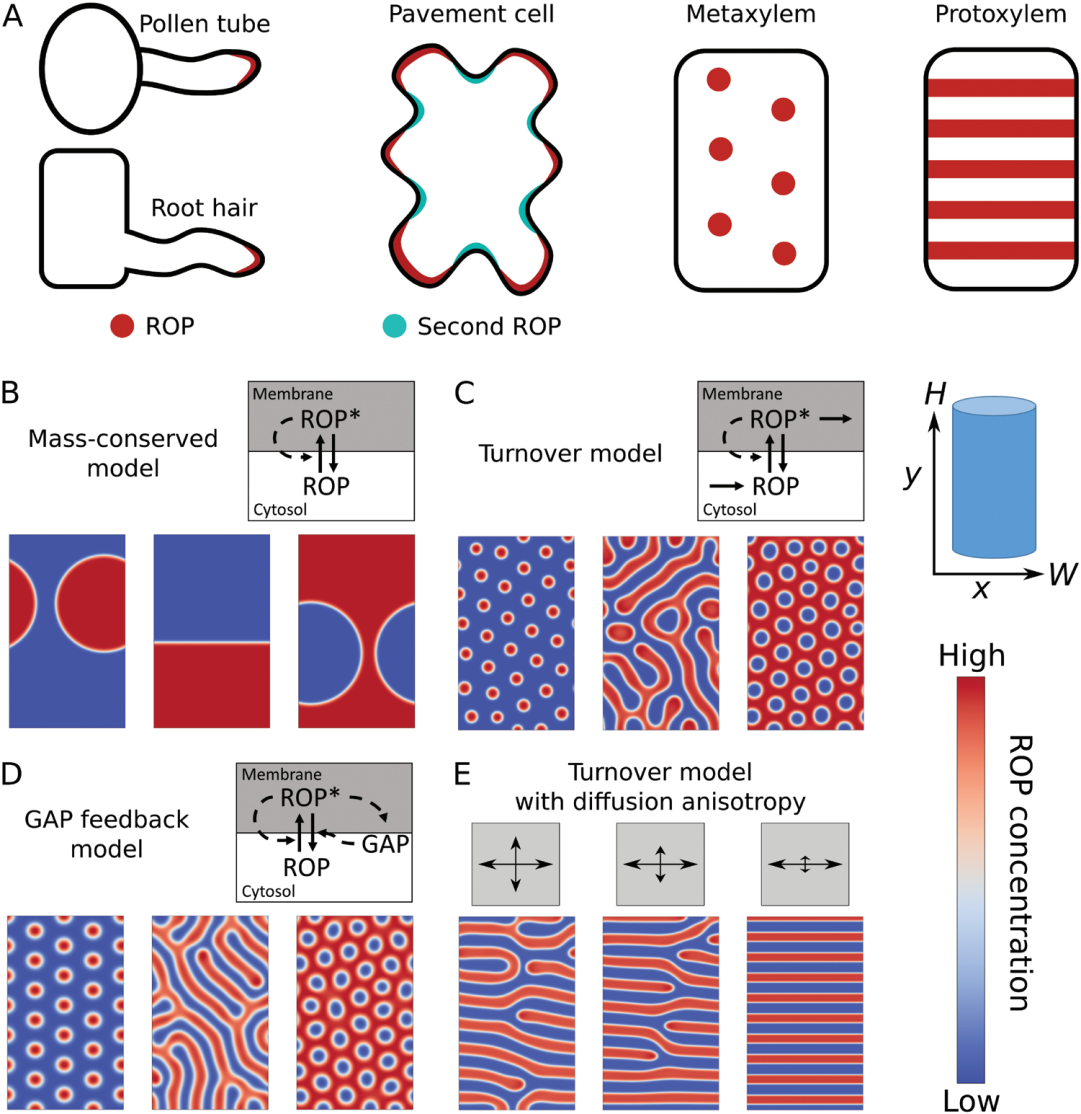
Patterns of ROP GTPase activity and model simulations that reproduce them. (A) In pollen tubes and root hairs, the growth response is dominated by a single cluster of active ROP at the growing tip, while developing pavement cells, metaxylem, and protoxylem have regular patterns of ROP activity, with pavement cells showing complementary activity of antagonistic ROPs and protoxylem showing an oriented ROP pattern. (B–E) Models for ROP GTPase patterning produce different types of patterns. (B) A mass-conserved model (no production or degradation), in which active ROP (ROP*) promotes activation of inactive ROP, always yields a single cluster of active ROP. (C) Adding turnover through production of an inactive form and degradation of an active form allows for the formation of regular patterns with multiple clusters. (D) A similar effect can be achieved by adding negative feedback to the mass-conserved model, where the active ROP activates GAP, which in turn deactivates ROP. (E) A specific orientation can be imposed on a regular pattern by restricting active ROP diffusion in one direction during the pattern formation process. (B–D) were adapted from Jacobs *et al.* (2019) and (E) from Jacobs *et al.* (2020).

Small GTPases are deeply conserved molecular switches that cycle between an active, GTP-bound, and an inactive, GDP-bound, state. In their active state, small GTPases can interact with various so-called effectors, locally inducing their activity (Nagawa *et al.*, 2010; Cherfils and Zeghouf, 2013; Feiguelman *et al.*, 2018; Ou and Yi, 2022). The Rho-family of small GTPases is involved in many signalling processes related to subcellular domain formation (Etienne-Manneville and Hall, 2002; Fritz and Pertz, 2016). In plants, plasma membrane patterning is governed by a single subfamily, called ‘Rho of Plants’ (ROP) or similar (Nielsen, 2020). ROPs can form a large variety of patterns of active ROP, used to specify the location of different cellular processes (Fig. 1A). These processes range from the initiation of root hairs (Denninger *et al.*, 2019) and the maintenance of a polar growth tip on root hairs and pollen tubes (Li *et al.*, 1999; Molendijk *et al.*, 2001; Li *et al*., 2023), to the establishment of lobes/indents on leaf pavement cells (Fu *et al.*, 2002, 2005, 2009) and the striped (Brembu *et al.*, 2005) and spotted (Oda *et al.*, 2010; Oda and Fukuda, 2012, 2013) patterns that inform the intricately patterned secondary cell wall depositions in primary xylem—in interaction with cortical microtubules (Oda *et al.*, 2010; Schneider *et al.*, 2021). All of the above examples lead to changes in cell shape and/or cell wall structure, which is reflected in the large number of ROP effectors that affect the actin or microtubule cytoskeleton (Feiguelman *et al.*, 2018). Where the first two examples require a single spot of active ROP, the latter two require the stable coexistence of a large number of active ROP clusters. Notably, both the single-cluster pattern of root hair initiation and the multi-cluster pattern of leaf pavement cells are governed by AtROP2/4/6 (Fu *et al.*, 2002, 2005, 2009; Smokvarska *et al.*, 2021), demonstrating that the difference between these patterning outcomes is not primarily determined by the specific ROP(s) involved and must happen via other proteins or biochemical ‘tuning parameters’: plants can vary which specific proteins regulating ROP activity (see below) are used in a particular process, as well as the amounts and ratios of the different proteins involved.

## ROP patterning by the text book

**Fig. 2.**
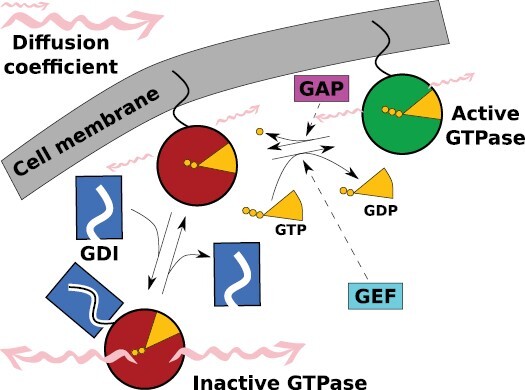
The ‘standard picture’ of ROP patterning. ROP cycles between the inactive, GDP-bound (red), and active, GTP-bound state (green). ROP activation is promoted by GEF and inactivation by GAP. Inactive ROP is reversibly removed from the cell membrane by GDI (blue box), which shields its hydrophobic tail. Diffusion in the cytosol is faster than at the membrane, as indicated with wiggle-arrows of different sizes (not to scale). Figure adapted from Jacobs (2022).

In the standard picture (Fig. 2) (Cherfils and Zeghouf, 2013; Fehér and Lajkó, 2015; Feiguelman *et al.*, 2018), activation of ROPs is catalysed by ROP-guanine nucleotide exchange factors (GEFs) whereas GTPase-activating proteins (GAPs) stimulate the GTPase activity that leads to the inactivation of ROPs. Guanine nucleotide dissociation inhibitors (GDIs) are involved in the temporary release of the inactive form from the membrane by shielding the hydrophobic tail, a process referred to as GDI cycling. So, in the standard picture, active ROP is membrane bound and inactive ROP is cytosolic. This results in a much higher diffusion coefficient for inactive ROP (Postma *et al.*, 2004; Fritz and Pertz, 2016), which is one of the critical ingredients of pattern formation in reaction–diffusion mechanisms (Turing, 1952; Gierer and Meinhardt, 1972). All mathematical models describing ROP patterning use such a mechanism (Goryachev and Leda, 2020). The precise details of the molecular interactions and their translation into mathematical form determines whether a single cluster is formed regardless of domain (cell) size, or if multiple clusters can stably coexist (Fig. 1B–D) (Jacobs *et al.*, 2019; Goryachev and Leda, 2020). Active ROP interacts with various effectors, many of which affect the cytoskeleton (Feiguelman *et al.*, 2018). In this way, ROPs can modify the cell shape of growing cells and cell wall anisotropy. Patches of active ROP can induce local cell expansion via the recruitment of actin and the resulting local exocytosis of cell wall material (Fu *et al.*, 2002; Gu *et al.*, 2003; Smokvarska *et al.*, 2021). With the help of different effectors, patches of active ROP can change cell wall anisotropy and secondary cell wall deposition through locally stimulating or depolymerizing the cortical microtubules (Fu *et al.*, 2009; Oda and Fukuda, 2012), which act as ‘railroad tracks’ guiding the deposition of cellulose microfibrils (Paredez *et al.*, 2006; Gutierrez *et al.*, 2009; Chan and Coen, 2020).

## Models for small GTPase patterning

### Generic mathematical small GTPase models

Before ROPs were addressed, mathematical models have been developed for small GTPase patterning in budding yeast and animal (keratocyte) cells (Marée *et al.*, 2006; Otsuji *et al.*, 2007; Goryachev and Pokhilko, 2008; Mori *et al.*, 2008; Jilkine and Edelstein-Keshet, 2011). A typical example of a small GTPase patterning model, which is also used for ROPs, is shown below. It was originally developed for understanding animal cell polarization (Mori *et al.*, 2008, 2011). This model consists of two partial differential equations (PDEs; see Box 1 for an overview of selected terminology), one for active (*u*) and one for inactive GTPase (*v*). Both components diffuse, with a larger diffusion coefficient for inactive GTPase owing to GDI cycling $\left(D_{v}\gg D_{u}\right)$. The mathematical structure of this model is depicted in Fig. 1B, along with its default behaviour in the patterning regime.

Box 1. **Terminology**

**ODE/PDE**: ordinary/partial differential equation.

**Deterministic PDE framework**: a deterministic (‘normal’) PDE model always yields exactly the same outcome if the initial condition and model parameters are exactly the same. This is not the case in a stochastic PDE framework, in which random noise is added at every time step.

**Regular pattern**: a pattern of evenly spaced clusters of ROP activity (e.g. a spotted or striped pattern).

**Competition**: model outcome where clusters of active ROPs compete with each other until only a single cluster remains.

**Coexistence**: model outcome where a stable pattern of regularly spaced clusters of ROP activity is established (e.g. a spotted or striped pattern).

**Quasi-coexistence**: model outcome where multiple clusters of active ROP can coexist for a long time, but which ultimately behaves the same as competition.

**Mass-conserved model**: model in which the total amount of ROP does not change (i.e. ROPs may be activated and inactivated, but not produced or degraded).

**Phase separation process** (this context): in a phase separation process, two phases such as oil and water demix, thereby reducing the (oil–water) interface between the two phases. Mathematically, the competition case of ROP models behaves the same. In this case, the length of the interface or boundary between areas of low and high levels of active ROP decreases over time to a (local) minimum.

$$\begin{array}{l} \frac{\partial u}{\partial t}=f\left(u,v\right)+D_{u}\nabla^{2}u \end{array}$$

$$\begin{array}{l} \frac{\partial v}{\partial t}=-f\left(u,v\right)+D_{v}\nabla^{2}v. \end{array}$$ (1)

Importantly, this model is mass conserved, meaning that GTPase is converted between active and inactive forms as described by the function *f* (*u*,*v*), but never degraded or produced. A typical example of *f* (*u*,*v*) is:


$$\begin{array}{l} f\left(u,v\right)=bv+\gamma \frac{u^{2}}{K^{2}+u^{2}}v-\delta u, \end{array}$$ (2)

with *b* the (typically small) baseline activation rate, γ the maximum extra activation rate due to active GTPase feedback, *K* the concentration of *u* at which the feedback activation is half of the maximum rate, and δ the constant inactivation rate. The conversion function is non-linear and has two important effects: it provides a positive feedback on activation that is critical for clustering, and the activation rate saturates, which limits the maximum concentration of active GTPase to a flat plateau level. At the scale of standard confocal microscopy, this would show as a roughly homogeneous concentration within an active GTPase cluster, which rapidly increases from background level at the edges.

Of course, multiple model variants exist. Some models include more than two states of GTPase, for example including GEF- and GAP-bound states (Goryachev and Pokhilko, 2008; Nagashima *et al.*, 2018), or important effectors (Grieneisen, 2009; Champneys *et al.*, 2021). Some models for cell polarization do not impose saturation on the self-activation, resulting in a faster establishment of a unipolar pattern (Goryachev and Pokhilko, 2008; Goryachev and Leda, 2020). Another modification of the positive feedback is to have multiple GTPases that mutually inhibit each other. The resulting double negative feedback loop fulfils the same role as the direct positive feedback loop in Equation 2, and the model produces similar patterns (Grieneisen, 2009; Holmes and Edelstein-Keshet, 2016; Jacobs *et al.*, 2019). Some modifications qualitatively impact model behaviour, including explicit turnover (Fig. 1C) (Verschueren and Champneys, 2017; Jacobs *et al.*, 2019), negative feedback through GAP activation by active GTPase (Fig. 1D) (Jacobs *et al.*, 2019; Herron *et al.*, 2022), and an inactive GTPase state that cannot directly be activated (Chiou *et al.*, 2021). We address the underlying mechanisms later in this review.

### Plant-specific aspects of ROP models

Plant cells differ from animal and yeast cells in more than the names of the small GTPases. This is also reflected in dedicated ROP models. The cortical microtubule array is one such distinguishing feature, which could make the diffusion of membrane-bound ROP anisotropic (replacing *D*_*u*_ in Equation 1 by a diffusion tensor and slightly modifying the equation accordingly) (Oda and Fukuda, 2012; Jacobs *et al.*, 2020). We address this further later in this review. Another plant-specific aspect is that ROPs can be activated by the plant hormone auxin, which is demonstrated for multiple ROPs and occurs within minutes (Xu *et al.*, 2010; Platre *et al.*, 2019). Indeed, several models exist in which the ROP activation rate (similar to the γ in Equation 2) explicitly depends on the local auxin concentration at the membrane (Grieneisen, 2009; Payne and Grierson, 2009; Breña Medina and Champneys, 2014; Breña Medina *et al.*, 2014; Avitabile *et al.*, 2018). In these models, auxin serves to coordinate ROP patterns between neighbouring cells (Grieneisen, 2009) or position the active ROP maximum (for a root hair) along a static intracellular gradient (Payne and Grierson, 2009; Breña Medina and Champneys, 2014; Breña Medina *et al.*, 2014; Avitabile *et al.*, 2018). Critically, plant cells must be able to sense these gradients along the membrane. After a controversial history (Napier, 2021), auxin-binding protein 1 (ABP1) may fulfil this role after all, together with transmembrane kinase 1 (TMK1) (Xu *et al.*, 2014; Friml *et al.*, 2022).

## Diversity in the ROP system

### Many ROPs and regulatory proteins

Despite the habit of mathematicians to treat all ROPs similarly, if not totally equivalently, the model plant *Arabidopsis thaliana*, for example, has 11 ROPs, 15 GEFs [SPIKE1 (Qiu *et al.*, 2002) and 14 so-called PRONE-domain-containing ROPGEFs (Berken *et al.*, 2005; Gu *et al.*, 2006)], nine GAPs [of which six are ROPGAPs (Wu *et al.*, 2000) and three are PHGAPs or RENGAPs (Stöckle *et al.*, 2016)], and three GDIs (Feiguelman *et al.*, 2018; Nielsen, 2020), and employs different ROPs, etc. in different contexts (Feiguelman *et al.*, 2018; Smokvarska *et al.*, 2021). Therefore, different regulatory proteins could be employed in tuning the behaviour of the ROP system in different developmental contexts. It turns out, for example, that GEFs and GAPs can show high specificity for a particular ROP in pavement cells, a system that is simultaneously controlled by multiple ROPs (Igisch *et al.*, 2022). Also, differences in biochemical parameters such as mobility and ROP inactivation rate among GAP types can lead to different effects on the plant phenotype, as recently observed in the moss *Physcomitrium patens* (Ruan *et al.*, 2023).

Furthermore, the mathematical models only yield patterns in specific parameter regimes (Goryachev and Pokhilko, 2008; Holmes and Edelstein-Keshet, 2016; Jacobs *et al.*, 2019; Goryachev and Leda, 2020; Champneys *et al.*, 2021), meaning that besides the set of specific of ROPs and regulatory proteins, their amounts also matter. For example, different expression systems may yield different amounts of the relevant proteins, and only some of these may happen to fall in the proper patterning regime, which may explain why different research groups found different GEFs with which AtROP11 would form patterns (Oda and Fukuda, 2012; Nagashima *et al.*, 2018; Sternberg *et al.*, 2021).

ROPs are sometimes activated in response to other signalling cascades, for example involving receptor-like kinases at the membrane, such as TMK1 activated in response to auxin (Xu *et al.*, 2014; Yu *et al.*, 2022). Such signals may be localized to or stronger at specific sides of the cell. It is likely that this could set the position of the (first) active ROP cluster.

### A type-I/II split in the ROP family

ROPs can be divided into three clades based on the similarity of their nucleotide sequences (Fowler, 2010) or two types based on the similarity of the amino acid sequence of their C-terminal hypervariable region (Winge *et al.*, 1997). Of these, the distinction between type-I and type-II ROPs seems particularly functionally relevant, because the hypervariable region is involved in membrane insertion. Type-I ROPs have animal and fungal orthologues, whereas type-II ROPs are plant specific. The model plant *A. thaliana* has eight type-I ROPs (AtROP1–AtROP8) and three type-II (AtROP9, 10, and 11), but numbers of ROPs and the ratio between type-I and II vary substantially within the plant kingdom (Fowler, 2010).

**Fig. 3.**
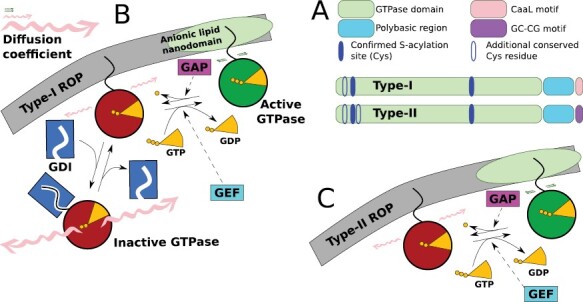
Type-I and type-II ROPs. (A) Overview of the molecular differences between type-I and type-II ROPs and their potential consequences. The key difference between type-I and type-II ROPs is the C-terminal motif that is important for membrane interaction. These motifs undergo different post-translational modifications (see text). Additionally, type-II ROPs contain one more conserved Cys residue in the GTPase domain. For supporting multiple sequence alignment, see Supplementary Fig. S1. (B and C) Possible consequence of these differences: it is suggested that only type-I ROPs undergo GDI cycling (Sternberg *et al.*, 2021), which would imply that type-I ROPs have three intrinsic levels of diffusion (active, inactive-membrane bound, and inactive-free) and type-II ROPs have only two. Formation of ‘nanoclusters’ has been observed for various type-I and type-II ROPs (Platre *et al.*, 2019; Fuchs *et al.*, 2021, Preprint; Smokvarska *et al.*, 2021; Sternberg *et al.*, 2021), but preferred lipid compositions probably differ among ROPs (Hirano *et al.*, 2018). The magnitude of typical diffusion coefficients for the different states is indicated with wiggle-arrows of different sizes (not to scale).

Specific lipid modifications are critical for membrane interaction. Type-I ROPs have a C-terminal CaaL motif at which they are prenylated, cleaved, and methylated, increasing tail hydrophobicity and, hence, membrane affinity (Young *et al.*, 2001; Bracha-Drori *et al.*, 2008; Sorek *et al.*, 2011). Type-II ROPs, in contrast, are *S*-acylated at a C-terminal GC–CG box motif before additional modifications (Lavy and Yalovsky, 2006; Bracha-Drori *et al.*, 2008). The two types of ROP probably also differ at another kind of post-translational modification: ROPs are transiently *S*-acylated at conserved Cys residues in the GTPase domain upon activation, which, at least in AtROP6, is required for active ROP accumulation in nanodomains (Sorek *et al.*, 2017; Smokvarska *et al.*, 2020). Type-I ROPs contain three relevant conserved Cys residues. The same residues are conserved in type-II ROPs, which, moreover, contain one more conserved Cys (Fig. 3A; Supplementary Fig. S1). Consequently, type-II ROPs could have more extreme differences in the chemical properties of active versus inactive GTPase domains.

Clusters of different ROPs may be reinforced through the local enrichment of specific anionic phospholipids. For example, a phosphatidylinositol-4,5-bisphosphate [PtdIns(4,5)P_2_]-enriched AtROP2 (type-I) domain exists at the tip of a growing root hair, and a PtdIns(3,5)P_2_-enriched AtROP10 (type-II) domain at the shank (Hirano *et al.*, 2018). Further research is needed, however, to establish whether such different affinities follow the type-I/II split.

### Multiple options for faster diffusion of inactive ROP

A consequence of the different C-terminal modifications may be that type-II ROPs are not subject to GDI cycling. RhoGDI1, at least, interacted with the type-I AtROPs 2, 4, and 6, but not with any of the type-II AtROPs in a yeast two-hybrid assay (Sternberg *et al.*, 2021), and the authors claim that a similar situation is likely to hold for the other GDIs based on their structural and functional conservation (Carol *et al.*, 2005; Sun *et al.*, 2015; Sternberg *et al.*, 2021). This claim requires further experimental confirmation, but if GDI cycling is indeed restricted to type-I ROPs, and no other proteins turn out to exist that fulfil a similar role for type-II ROPs, then it has serious consequences for the theoretical foundation of the standard ROP model. All the mathematical models require that the diffusion of inactive ROP is sufficiently faster than that of active ROP (i.e. $D_{v}\gg D_{u}$ in Equation 1) (Goryachev and Leda, 2020), and the required parameter difference is typically justified by GDI cycling (Marée *et al.*, 2006; Goryachev and Pokhilko, 2008; Mori *et al.*, 2008; Grieneisen, 2009; Payne and Grierson, 2009; Breña Medina *et al.*, 2014; Jacobs *et al.*, 2019).

There is more, however, that can cause a difference in ROP diffusion. In animals, up to six different levels of diffusivity in Rho-like small GTPases have been reported, the lowest of which is orders of magnitude smaller than the others (Koo *et al.*, 2015). At the membrane, ROP diffusivity can be drastically reduced by ROP recruitment into anionic lipid nanodomains with a diameter of 50–70 nm (Smokvarska *et al.*, 2021). In plants, this was first observed for auxin-dependent activation of type-I AtROP6 and depends strongly on the relatively abundant anionic phospholipid phosphatidylserine (Platre *et al.*, 2019). Constitutively active AtROP6 accumulates more strongly in such nanodomains and its measured diffusion is more strongly reduced than wild-type AtROP6, which can also occur at the membrane in the inactive form (Smokvarska *et al.*, 2020). Nanodomains of (presumed) active ROP are also observed for numerous other ROPs, including type-II ROPs (Fuchs *et al.*, 2021, Preprint; Sternberg *et al.*, 2021), although activity is not always as carefully checked as with AtROP6. This implies that a difference in diffusivity of active and inactive ROP ($D_{v}\gg D_{u}$ in Equation 1) can be biochemically justified for all ROPs, even if some ROPs would indeed not undergo GDI cycling (Fig. 3B, C). As cytosolic diffusion (i.e. with GDI cycling) typically is faster than ‘free’ membrane-bound diffusion, however, the predominant underlying mechanism probably affects the spacing of coexisting clusters. Visual inspection of the figures in Sternberg *et al.* (2021) appears to weakly support the idea that type-I ROPs would produce more distantly spaced active ROP clusters than type-II ROPs when combined with the same GEF and GAP proteins, although a general claim would require further quantitative investigation. Additionally, in some cases where the underlying ROP system is tuned for coexistence (see below), loss of GDI cycling could make the difference between a single cluster or multiple clusters fitting on the (normal cell sized) domain. An observation that fits this scenario is that in the AtRhoGDI1 mutant *supercentipede1* (*scn1*), type-I AtROP2 accumulates in multiple root hair initials per cell rather than in the usual single cluster (Carol *et al.*, 2005).

It is important to note that the addition of GFP or similar tags, at both the N- and C-terminus, can interfere with ROP function and/or dynamic behaviour (e.g. Cheng *et al.*, 2020). Data without demonstration that the specific fluorescently tagged ROP construct fully complements the respective null mutant should, therefore, be treated sceptically.

Another interesting problem is the formation of the nanodomains themselves. In principle, the model in Equation 1 could be scaled to any size. The relevant question is whether patterns can be obtained at the relevant scale with realistic diffusion coefficients (i.e. whether the patterning could be achieved entirely by the ROP dynamics) or if there could be a different explanation. As a first-order approximation, the pattern size scales with the square root of the diffusion coefficients involved. All else being equal, a pattern with a 10 μm length scale (pavement cell lobes) requires 400× larger diffusion coefficients than a pattern with a 500 nm length scale (ROP nanoclusters; both length scales by approximation from data in Platre *et al.*, 2019 and Pan *et al.*, 2020). Diffusion coefficients of the type-I AtROP6 have been measured at ~0.1–1 μm^2^ s^–1^ for mobile populations and ~0.001–0.1 μm^2^ s^–1^ for immobile populations (Platre *et al.*, 2019; Smokvarska *et al.*, 2020), similar to Rac1 in animals (Das *et al.*, 2015), so the required difference in diffusion coefficients may just be realistic. Alternatively, mechanical effects via altered membrane curvature of the nanodomains (as best described for the animal small GTPase Ras) can fulfil a similar role and have their own range of feasible length scales (Semrau and Schmidt, 2009; Ogunyankin *et al.*, 2013; Veerman *et al.*, 2021). In either case, describing both the nanodomain pattern and the larger scale pattern at the same time is an intriguing prospect that would require a more complex description than a simple two-state model.

## Competition, coexistence, and quasi-coexistence in mathematical models

All GTPase models currently used are reaction–diffusion models of the substrate depletion type (Turing, 1952; Gierer and Meinhardt, 1972). These models have a regime in which the local depletion of substrate, namely inactive GTPase, surrounding a patch of active GTPase, results in an inhomogeneous distribution of active GTPase: the membrane pattern. In the parameter regime where patterns occur, two major patterning outcomes are possible for reaction–diffusion models. We refer to these as ‘competition’ and ‘coexistence’, but other terms are also used by different authors (Jacobs *et al.*, 2019; Goryachev and Leda, 2020; Chiou *et al.*, 2021). In the case of competition, ultimately only a single cluster of active GTPase remains regardless of domain size (Fig. 1B), whereas in the case of coexistence, multiple clusters can stably coexist (Fig. 1C, D). Some authors suggest that a subset of the competition cases can be used to describe multi-clustered patterns in biology, if the time scale of the competition between the last remaining clusters is so slow that multiple clusters coexist for the biologically relevant time scale (Chiou *et al.*, 2018). We here refer to this option as ‘quasi-coexistence’. For a mathematically sound but still accessible review about competition or coexistence for two-state (i.e. only describing the active and inactive form of a single GTPase) mass-conserved models of a single GTPase, we refer the reader to Goryachev and Leda (2020). Due to the restrictive choice of models discussed, that review mostly addresses the difference between competition and quasi-coexistence.

### Simple mass-conserved models show competition behaviour, but plants also require coexistence

**Fig. 4.**
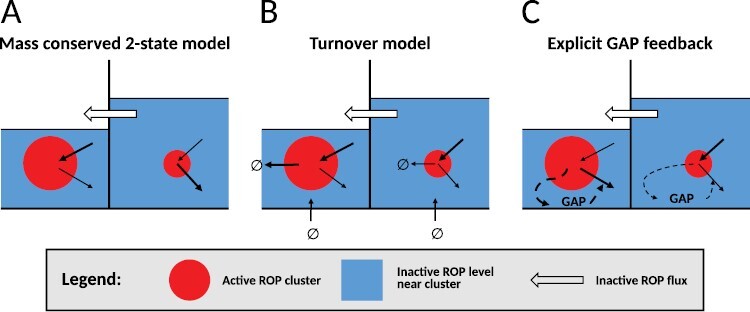
Cluster-level fluxes explain the difference between competition and coexistence. Under the standard (mass conserved two-state) model (A), ROP activation is proportional to total active ROP in the cluster (cluster area), whereas active ROPs are lost from the cluster at a rate proportional to cluster circumference. This balance is more favourable for larger clusters, resulting in a lower concentration of inactive ROP (blue ‘liquid levels’) surrounding the larger cluster and, therefore, a net flux of ROP from the smaller to the larger cluster, ultimately completely draining the smaller cluster. In the turnover model (B), all these fluxes remain, but these are balanced by degradation of active ROP proportional to total active ROP in the cluster and a homogeneous production of new (inactive) ROP independent of cluster size. With explicit GAP feedback (C), GAP activity and, hence, the ROP inactivation rate are larger in larger clusters, balancing the size advantage of the mass-conserved two-state model. Figure adapted from Jacobs *et al.* (2019).

The commonly used mass-conserved substrate depletion models (Mori *et al.*, 2008; Walther *et al.*, 2012; Holmes and Edelstein-Keshet, 2016; Chiou *et al.*, 2018; Brauns *et al.*, 2020), for example like Equation 1, all result in competition between clusters (Fig. 1B) (Jacobs *et al.*, 2019; Goryachev and Leda, 2020). This competition occurs because smaller clusters lose relatively more active GTPase through diffusion across their boundary, and larger clusters more effectively deplete the inactive GTPase supply, resulting in a diffusive flux of inactive GTPase from smaller to larger clusters (Fig. 4A) (Jacobs *et al.*, 2019). Mathematically, this process is similar to that of phase separation (Tateno and Ishihara, 2021). Since many biological processes produce multiple coexisting GTPase clusters, this model behaviour has sparked a search for mechanisms that allow multiple coexisting clusters.

One proposed solution is that in certain parameter regimes, the competition between two similarly sized clusters could be so slow that they coexist for biologically relevant time scales (Chiou *et al.*, 2018). Although this may work to explain spurious bud formation in certain yeast mutants (Goryachev and Leda, 2020), this quasi-coexistence can only reproduce phenomena with a low number of clusters (usually two), which, moreover must form simultaneously during *de novo* pattern formation. Splitting of clusters, as would, for example, be required for branching in tip-growing cells such as fungal hyphae (Harris, 2008, 2011; Arkowitz and Bassilana, 2015), or Arabidopsis root hairs in GEF3 or GEF4 overexpression lines (Denninger *et al.*, 2019), is impossible in such a model. Regular patterns with many clusters, moreover, such as those observed in leaf pavement cells and developing primary xylem, are not at all possible with such a mechanism. Also in animals, many-clustered small GTPase patterns occur, for example underlying the actin rosettes in frustrated phagocytosis (Herron *et al.*, 2022). Another process that is impossible with quasi-coexistence is the appearance of additional clusters as the domain grows. Such a process can be inferred, for example, from the observed cell shape changes of growing leaf pavement cells (Sánchez-Corrales *et al.*, 2018; Sapala *et al.*, 2018). Therefore, for many small GTPase patterning processes, especially in plants, the competition models are insufficient and models that produce true coexistence are required.

### Multiple options for coexistence

The mathematically simplest solution for obtaining true coexistence is the addition of turnover through the (linear) breakdown of active GTPase and spatially homogeneous production of new inactive GTPase (Fig. 1C) (Verschueren and Champneys, 2017; Jacobs *et al.*, 2019; Liu *et al.*, 2021). This immediately turns the produced patterns into the familiar-looking Turing patterns of regularly spaced spots, stripes, or gaps (Meinhardt, 2012). The addition of turnover achieves this coexistence by providing new inactive GTPases homogeneously while removing active GTPases at a rate proportional to cluster size, thus balancing the size advantage (Fig. 4B) (Jacobs *et al.*, 2019). Mathematically, this can be described as an interrupted phase separation process (Brauns *et al.*, 2021). Note that this mechanism requires active GTPase degradation—from the membrane—not just inactive GTPase degradation (Jacobs *et al.*, 2019). Biologically, this would imply that there must be either direct degradation of the active GTPase at the membrane, or some form of sequestering that ultimately leads to degradation, without the GTPase first becoming available in its inactive form.

A different option for stable coexistence is the explicit addition of a negative feedback through a set of GAP protein equations (Fig. 1D) (Jacobs *et al.*, 2019). In this mass-conserved model, active ROP activates GAP, which in turn promotes local ROP inactivation. Although all terms for GAP in this model are linear, the faster diffusion of GAP than active ROP (supported by, for example, Sternberg *et al.*, 2021) makes the inactivation rate higher in larger clusters (Fig. 4C). In this way, the GAP equations introduce a negative feedback on cluster size, allowing for stable coexistence even if the GTPase turnover is negligible in the particular context (Jacobs *et al.*, 2019).

Yet another option for stable coexistence, that was introduced as a theoretical exercise, is the introduction of an inactive substrate state, such that a ROP molecule cannot immediately be reactivated upon inactivation, but has to be recharged first (Chiou *et al.*, 2021). In that way, ROP molecules are effectively redistributed over the cell after inactivation, much like with the turnover mechanism (Jacobs *et al.*, 2022).

Note that models that are mathematically wired for true coexistence can still produce only a single cluster if only a single repetition of the pattern (e.g. one cluster) fits on the domain. This is, for example, the default outcome of the root hair positioning model (Payne and Grierson, 2009; Breña Medina and Champneys, 2014; Breña Medina *et al.*, 2014; Avitabile *et al.*, 2018), which has turnover and is capable of coexistence, but produces a single root hair spot under conditions representing a normal root hair-forming cell (Breña Medina *et al.*, 2014; Avitabile *et al.*, 2018). Also, experiments suggest that this hidden coexistence regime applies to root hairs, as GEF4 overexpression results in root hair branching and GEF3 overexpression results in the initiation of multiple root hairs on a single cell as well as root hair branching (Denninger *et al.*, 2019). The primary effect of GEF overexpression would be an increase of γ in Equation 2. This parameter change indeed reduces the (minimum) distance between spots (Supplementary Fig. S3), so could explain the experimental observations.

### Modelling choices may falsely suggest quasi-coexistence

Simulations are often used in determining the pattern outcome of a particular ROP model. The simulation of PDEs, however, is computationally demanding and, consequently, authors often restrict their simulations to one dimension (1D). In the case of ‘competition’, however, this can produce misleading results. The competition process is in essence a phase separation process, and a key driver of phase separation is the reduction of total interface length (Rubinstein and Sternberg, 1992). In 1D, the interface ‘length’ depends only on the number of clusters, whereas in 2D, the size of the clusters also matters. Consequently, phase separation occurs faster in 2D than in 1D (Ishihara *et al.*, 2007; Jacobs *et al.*, 2019). For example, the original mass-conserved model of Mori *et al.* (2008) in 1D shows multiple clusters that appear stable for long simulation times (in the relevant parameter regime) (Walther *et al.*, 2012), but the time until a single cluster dominates the competition is much shorter in 2D (Jacobs *et al.*, 2019). As a general lesson, results from 1D models, particularly simple forward simulation results, provide poor estimates of the time scales of multi-cluster pattern stability.

Another potential problem with the transient coexistence option is that the studies advocating this solution use the deterministic PDE framework (a.k.a. ‘normal’ PDEs). A single look at a microscopic image of a ROP cluster suffices to understand that this process is inherently noisy. Addition of such noise (i.e. switching to a stochastic PDE framework) makes interfaces such as the cluster edges move by Brownian motion and substantially speeds up the phase separation process (Funaki, 1995; Lee, 2018). So, the biological facts of the dimensionality of the cell membrane and the existence of molecular noise further limit the applicability of the slow competition scenario for biological phenomena that must be caused by two or (few) more active ROP clusters.

When the deterministic model is capable of forming stably coexisting clusters, however, the addition of noise preserves that possibility, although the relevant parameter regime may shift (Woolley *et al.*, 2017). Also in this case, noise tends to speed up the pattern formation process (Kim *et al.*, 2017).

### Biological questions from turnover models for coexisting ROP clusters

The fact that stable coexistence can be achieved via multiple mechanisms raises the question of which model components are favoured by molecular data. To start, the dramatic effect of adding turnover to ROP models raises the question: should this process regularly be included? A modelling study on keratocytes reveals how dramatic the effect could be, showing cells with GTPase ‘measles’ or spiral waves rather than a single polarized front (Liu *et al.*, 2021).

Biologically, the main difference between a mass-conserved and a corresponding turnover model is time. At the limit where ROP turnover approaches zero, the equations of the turnover model indeed converge to the mass-conserved model (Verschueren and Champneys, 2017). Measurements in mouse cells show half-lives in the order of 10–30 h for regularly methylated GTPases (Backlund, 1997; Bergo *et al.*, 2004; see also Supplementary Table S1), though it remains unclear how comparable the turnover is between animal and plant GTPases. On the one hand, this would lead to substantial protein turnover at the time scales of our coexistence examples. The development of first lobes and additional lobes on leaf pavement cells occurs while the leaf blade is expanding (Sapala *et al.*, 2018), a process that can span multiple days. Similarly, ROP patterns that inform xylem patterning are likely to fully establish within at most 6–11 h and remain stable for up to several days (Schneider *et al.*, 2021). On the other hand, the reported rates are much lower than the default value of the active ROP degradation parameter used in several modelling studies of 0.01 s^−1^ (Payne and Grierson, 2009; Breña Medina and Champneys, 2014; Breña Medina *et al.*, 2014), which yields a half-life of just over 1 min.

This discrepancy between (animal) data and model parameters probably has a large impact on the spacing of spots (Breña Medina *et al.*, 2014; Liu *et al.*, 2021). Therefore, obtaining a realistic cluster spacing for pavement cell or xylem patterning with a realistic turnover rate, and realistic values for all other parameters, would require a complete reparameterization of the model, which—to our knowledge—has not been attempted yet and may or may not be possible. If the model can be simulated fast enough, however, evolutionary algorithms could be very helpful in finding regimes producing the right pattern in such a large parameter space, as demonstrated for an animal GTPase model (Herron *et al.*, 2022).

Another important question for the turnover model is where the degradation, or at least the initiation of the process, takes place in reality, as the model requires for coexistence that primarily active ROP is degraded (Jacobs *et al.*, 2019). Unfortunately, very little is known about this. On the one hand, cytosolic ROP might be more exposed to proteasome degradation, which would only affect inactive ROP. We could not find any relevant measurements of this. On the other hand, membrane endocytosis followed by degradation would primarily affect active ROP under the (simplified) assumptions of the standard picture (Fig. 2). There may be weak support for this in specific cases, as a study in tobacco pollen tubes found a (minor) source of clathrin-independent endocytosis from the tip, which seemed primarily destined for degradation—although besides a majority route of clathrin-dependent endocytosis from the subapical region, which probably is targeted for membrane recycling (Moscatelli *et al.*, 2007). It remains to be seen, however, how specifically this pathway targets active ROP in reality and whether this is at all relevant for patterns with many clusters (pavement cells, xylem).

The turnover model uses a spatially homogeneous production of inactive ROP. It is impossible that this assumption is met with mathematical precision, but an analysis of ROP fluxes at the cluster level (Jacobs *et al.*, 2019) suggests that this is not a problem, as long as the supply of new ROP to the membrane can be considered homogeneous with respect to the typical spacing of the active ROP clusters and is independent of the local/regional concentration of active ROP. With translation and post-translational processing at the endoplasmic reticulum (Bracha-Drori *et al.*, 2008), this condition is likely to be met in many cases. It may be, though, that the supply of ROP to the membrane becomes heavily biased in (tip)growing cells, as assumed, for example, in the tip growth modelling studies by Luo *et al.* (2017) and Li *et al.* (2018). If that is the case, the bias in ROP delivery to the membrane could contribute to localizing a ROP cluster (e.g. to the growing tip area) and suppressing cluster formation in other areas of the cell.

### Biological questions from GAP feedback models for coexisting ROP clusters

The GAP models also raise multiple questions. First, with respect to enabling coexistence, Jacobs *et al.* (2019) has already pointed out that this mechanism only appears to generate coexisting clusters when (active) GAP diffuses faster than active ROP. The reason for this appears to be that slowly diffusing active GAPs mostly affect the cluster where they originated, and therefore are more likely to prevent any clusters from forming than to promote cluster coexistence (Jacobs *et al.*, 2019). Although we are not aware of any measurements of GAP diffusion in the membrane (e.g. similar to the single molecule approach used for multiple ROPs; Platre *et al.*, 2019; Fuchs *et al.*, 2021, Preprint), the homogeneous cellular localization pictures in, for example Sternberg *et al.* (2021), are what is expected with a relatively high diffusion coefficient of (some form of) GAP. Ruan *et al.* (2023) shows more punctate PpRopGAP1 features than PpROP4 in moss, but on kymographs the RopGAP features appear far more mobile than those of ROP4 or GEF, again suggesting a larger effective diffusion coefficient for GAP. These measurements do not specifically concern active GAP, but if that form would have a similarly low diffusion coefficient to active ROP, that would probably show as local enrichment of total GAP near active ROP clusters. GAP (AtREN1) is also less confined to the plasma membrane than active ROP (AtROP2) (Kulich *et al.*, 2020).

Second, as GAPs are always involved, one may ask whether formation of a single cluster through a competition mechanism actually is possible, or if all instances of a single ROP or other small GTPase cluster are coexistence cases on a domain that is too small for an additional cluster. Here, it is important to note that GAPs actually are considered in all models via the linear inactivation rate, but the explicit GAP models introduce a GAP feedback through GAP activation proportional to the local ROP activity. It is this feedback that results in the switch to coexistence. Mere linear ‘GAP activity’ may be justified by rather homogeneous GAP distributions of fluorescently tagged GAP (Sternberg *et al.*, 2021), although these pictures do not inform about the critical element of the GAP models (Jacobs *et al.*, 2019): the activation status of GAP.

The considerations above raise a critical question: how is GAP activity itself regulated, and is this spatially correlated with active ROP clusters? GAPs contain various phosphorylation sites, and these could be used for regulation purposes. A very recent first study into PHGAP phosphorylation found that protein stability and, directly or indirectly, polar localization of AtPHGAP1 and 2 is regulated by [BR-INSENSITIVE (BIN2)-dependent] phosphorylation (Zhang *et al.*, 2022). The relevant sites, however, were in the C-terminal part and not in the GAP or PH-domains responsible for GAP activity. Along with other factors, this kind of regulation could translate to spatial variation in GAP activity along the membrane (Lauster *et al.*, 2022; Zhang *et al.*, 2022). This would, however, constitute a different kind of GAP feedback from that used in the coexistence models (Jacobs *et al.*, 2019).

Another highly relevant experimental observation for the GAP feedback model is that of ARMADILLO REPEAT ONLY (ARO) proteins. These ARO proteins bind PHGAP proteins and independently interact with ROPs; that is, they can bring the two together. In support of the model, ARO proteins bind more effectively to active ROP than to inactive ROP. Additionally, they may bind selectively to a subset of ROPs only (Kulich *et al.*, 2020). Notably, the *aro2/3/4* triple knockout mutant has a dramatic effect on root hairs and trichomes, but appears not to affect the puzzle shape of pavement cells (Kulich *et al.*, 2020), although PHGAP1 and 2 are required in that process (Lauster *et al.*, 2022; Zhang *et al.*, 2022). This raises the question of what enables coexistence in pavement cells, a system of mutually inhibiting ROPs. Is local GAP activity (also) tuned via a different mechanism, or is the pattern maintained by another mechanism for coexistence?

## Interaction with the cytoskeleton and dynamic cell geometry

For their effect on cell shape and secondary cell wall structure, ROPs must closely interact with the cytoskeleton. The impact of ROPs on the cytoskeleton is reflected in the large number of ROP effectors that affect the cytoskeleton, for example by changing actin or microtubule dynamics (Feiguelman *et al.*, 2018). This is not a one-way interaction, however. Changes in actin dynamics and subsequent changes in the actin cytoskeleton affect endo- and exocytosis, with possible effects on the supply of membrane proteins and the evolution of cell shape that are relevant for ROP patterning (Basu *et al.*, 2008; Luo *et al.*, 2017; Li *et al.*, 2018; Cheng *et al.*, 2020). Changes in microtubule dynamics can affect the growth anisotropy of the cell wall, indirectly, via their effect on the degree of alignment and other aspects of the organization of the cortical microtubule array (Ishida *et al.*, 2007; Deinum and Mulder, 2013; Lindeboom *et al.*, 2013, 2019; Vineyard *et al.*, 2013; Nakamura *et al.*, 2018), which guides the deposition of cellulose microfibrils (Paredez *et al.*, 2006; Gutierrez *et al.*, 2009; Chan and Coen, 2020). Microtubules can also more directly affect ROP patterning by making ROP diffusion at the membrane anisotropic (Fig. 1E) (Oda and Fukuda, 2012; Jacobs *et al.*, 2020).

The interaction of ROP patterning and cell growth has been modelled in the context of a (tip-growing) pollen tube (Luo *et al.*, 2017; Li *et al.*, 2018). This mass-conserved model does not use a full PDE for the amount of inactive ROP, but a single homogeneous cytosolic pool. Consequently (Jacobs *et al.*, 2019), this model always generates a single cluster of active ROP, which through positive feedback remains at the tip where it was initiated. It would be very interesting to couple a ROP model capable of stable or even quasi-stable coexistence to cell growth to investigate how evolution of pavement cell shape (Sánchez-Corrales *et al.*, 2018) and root hair branching in GEF overexpression lines (Denninger *et al.*, 2019) depend on the speed of cell growth. An earlier (static) pavement cell model lacked this stable coexistence behaviour (Grieneisen, 2009), but, as discussed above, multiple options for coexistence have been identified since then (Verschueren and Champneys, 2017; Jacobs *et al.*, 2019; Champneys *et al.*, 2021).

Primary xylem is a system where the joint patterning outcomes of ROPs and microtubules can be studied without cell shape changes. Various changes that specifically affect microtubules impact the patterns formed by the system as a whole. Xylem patterning is indeed affected by taxol treatment (Oda and Fukuda, 2012), loss of function of the microtubule-severing enzyme katanin (Schneider *et al.*, 2021), loss or overexpression of microtubule–membrane linker IQD13 (Sugiyama *et al.*, 2017), and changes in the level of microtubule-associated protein MAP70-5 (Pesquet *et al.*, 2010). Note, however, that these effects are most easily observed through changes in the pattern of secondary cell wall deposition, which follows the cortical microtubules (Schneider *et al.*, 2021). Where reported, however, ROP patterns do match the microtubule patterns.

### Case study: are type-I/II ROPs different in their potential for xylem patterning?

For microtubules to orient ROP patterns as observed during xylem patterning (Oda and Fukuda, 2012; Sugiyama *et al.*, 2017), they must act as diffusion barriers for ROPs (Jacobs *et al.*, 2020) (Fig. 1E). The existence of such a diffusion barrier effect has been inferred from localization patterns of the effector MIDD1 (Oda and Fukuda, 2012). Incorporating this effect via anisotropic diffusion in ROP models or similar demonstrates that co-alignment between microtubules and ROP patterns requires that the strongest diffusion anisotropy is on active ROP (Hiscock and Megason, 2015; Jacobs *et al.*, 2020). This condition is easily met with the help of GDI cycling, as cytosolic diffusion is hardly hindered by membrane-bound microtubules. If there indeed were no GDI cycling, and, in that case, a difference in diffusion of active and inactive ROP were achieved through trapping active ROPs in lipid nanodomains only, the existence of a distinct diffusion anisotropy for active and inactive ROPs would be much less obvious. Notably, the one ROP implicated from the metaxylem, the best studied primary xylem, is AtROP11, a type-II ROP (Oda *et al.*, 2010; Oda and Fukuda, 2012, 2013). All reported components including AtROP11 are highly expressed in the zone of protoxylem formation as well (Brady *et al.*, 2007; Winter *et al.*, 2007). So, what are the implications for xylem patterning if the suggestion that type-II ROPs do not undergo GDI cycling (Sternberg *et al.*, 2021) is indeed true?

It is likely that multiple ROPs are involved in the protoxylem, which forms banded patterns that follow the orientation of the initial microtubule array. The *AtROP11* loss-of-function mutant has "no clear protoxylem phenotype" (Oda and Fukuda, 2012). Additionally, a large number of ROPs are expressed in the zone of protoxylem formation (Brady *et al.*, 2007; Winter *et al.*, 2007) and the type-I AtROP7 has been observed in striated patterns during protoxylem development (Brembu *et al.*, 2005). In other words, GDI cycling can easily be returned into the protoxylem system via the involvement of one or more type-I ROPs.

This does not explain, however, why overexpression of the microtubule–membrane linker IQD13, which most probably leads to stronger anisotropic diffusion reduction, results in more elliptic co-aligned gaps in the metaxylem (Sugiyama *et al.*, 2017). This raises the question of what other options exist for coupling ROP and microtubule patterns.

Interestingly, barley contains a microtubule-associated GAP (HvMAGAP1) that restricts lateral ROP (HvRAC1) diffusion in the presence of cortical microtubules (Hoefle *et al.*, 2011, 2020). The C-terminal part of this MAGAP that contains the microtubule interaction domain is poorly conserved outside the monocots (Supplementary Fig. S2) (Hoefle *et al.*, 2011). It is not at all clear yet, therefore, that certain GAPs could offer the required coupling in dicots such as Arabidopsis.

Another option, one that is poorly explored experimentally, is that the relevant protein for translating anisotropy of the cortical microtubule array to the ROP pattern actually is GEF, not active ROP. The idea is supported by observation in root hair initiation that GEF3 serves as ‘a landmark protein’ for ROP at the root hair initiation domain (Denninger *et al.*, 2019) and GEF3 is less mobile in the root hair initiation domain than AtROP2 (Fuchs *et al.*, 2021, Preprint). Additionally, a parameter sweep in a six-component PDE model of ROP patterning in the metaxylem suggested that the slow diffusion requirement was strongest on the GEF–ROP complex, not on active ROP itself (Nagashima *et al.*, 2018).

Along the same lines, proteins other than the standard ROP signalling components could also be hypothesized to facilitate an interaction between ROPs and microtubules, for example, MAP70-5 that is located at the borders of microtubule bundles and gap boundaries during tracheary element differentiation (Pesquet *et al.*, 2010). This would introduce rather stable, discrete boundaries limiting ROP diffusion rather than a spatially homogeneous anisotropic diffusion reduction, and thus may work to co-orient ROP and microtubule patterns even in a system without GDI cycling.

## Concluding remarks: realistic behaviour versus realistic models

In this review, we have discussed multiple aspects of diversity in the proteins involved in ROP patterning in relation to mathematical models of the process. GAPs and GEFs, which regulate ROP activity, can have differential affinity for different ROPs and may themselves be inhomogeneously distributed (Kulich *et al.*, 2020; Fuchs *et al.*, 2021, Preprint; Igisch *et al.*, 2022; Lauster *et al.*, 2022); ROP patterns can be reinforced through changes in the local anionic lipid composition of the membrane (Hirano *et al.*, 2018); specifically microtubule-associated GAPs (at least in monocots) and other proteins can control the degree of ROP diffusion restriction by cortical microtubules (Pesquet *et al.*, 2010; Hoefle *et al.*, 2011, 2020; Sugiyama *et al.*, 2017), etc. A substantial part of the differences in behaviour that we described correlates with the biochemical differences between type-I and II ROPs. The suggestion that only type-I ROPs would undergo GDI cycling (Sternberg *et al.*, 2021), however, should be treated with caution until it is more firmly established experimentally. Notwithstanding, this suggestion is very interesting from a theoretical perspective, because it would affect the underpinning of the models, the interplay with the (microtubule) cytoskeleton, and the scale of patterns that are formed. Regardless of ROP type, the existence of more than two substantially different levels of ROP diffusivity (Koo *et al.*, 2015; Platre *et al.*, 2019; Smokvarska *et al.*, 2021) is mathematically interesting to explore. A more detailed, multi-state ROP model may also increase our understanding of the behaviour of dominant-negative ROP mutants, which are sometimes observed at the membrane and may interact poorly with GDI (Molendijk *et al.*, 2008; Sun *et al.*, 2015), and improve our interpretation of experiments with these mutants.

Investigating all this diversity is bound to come with computational challenges, especially when simultaneously considering cell growth and possible cell shape changes. Fortunately, the behaviour of the ROP patterning module always falls into one of a few generic classes: coexistence, competition, or quasi-coexistence, which can also be obtained with relatively simple two-state models (Mori *et al.*, 2008; Verschueren and Champneys, 2017; Jacobs *et al.*, 2019; Goryachev and Leda, 2020). In many cases, this, after initial verification, justifies the use of the computationally simplest option (e.g. Jacobs *et al.*, 2020). To what extent the gain in simulation time outweighs the less direct link with experiments will depend on the particular research question.

Finally, let the generic behaviour of reaction–diffusion models also serve as a warning, as obtaining the right pattern by itself provides no evidence that a specific model properly describes the underlying biology.

## Supplementary data

The following supplementary data are available at *JXB* online.

Table S1. Small GTPase protein half-lives and their dependence on methylation status (animal data).

Fig. S1. Multiple sequence alignment showing conserved Cys (C) residues in type-I and II

ROPs.

Fig. S2. Multiple sequence alignment showing BLAST hits to the C-terminal domain (residues 319-484) of HvMAGAP1.

Fig. S3. Steady-state patterns for different values of feedback activation parameter γ, generated by the wave pinning model with turnover (WPT), a model that allows coexisting clusters.

erad447_suppl_Supplementary_Data

## Data Availability

This review contains no new experimental data. Protein alignments supporting the claims in the main text are based on existing data available through NCBI. The alignments themselves are available in the supplementary data.
