# Supplementary material for: Rho of Plants patterning: linking mathematical models and molecular diversity
Source: J Exp Bot. 2023 Nov 14;75(5):1274–88. doi: 10.1093/jxb/erad447 (PMC10901209; doi:10.1093/jxb/erad447)
Supplement: erad447_suppl_Supplementary_Data [file erad447_suppl_supplementary_data.pdf]

Supplementary material for: ROP Patterning: Linking Mathematical Models and Molecular Diversity

Eva E. Deinum and Bas Jacobs
Journal of Experimental Botany

1 Supplementary table

Table S1: Small GTPase protein half-lives and their dependence on methylation status (animal data) Note that the methylation status of the C-terminal isoprenylcysteine is not an independent tuning parameter of protein half-life, as this methylation strongly affects the membrane affinity of the protein (Bracha-Drori et al., 2008) Methylation percentages, where measured, in brackets.

Protein(s)	Methylated	Unmethylated	Reference
Rho[1]	22 ± 9.4 h	2.8 ± 0.4 h	(Bergo et al., 2004)
Ras[1]	13.9 ± 6.1 h	32.5 ± 6.0 h	(Bergo et al., 2004)
RhoA[2]	31 h (>90%)	12 h (31%)	(Backlund, 1997)
RhoA	31 h (100% → 100%)	8.6 h (0%)	calculated from above[3]
RhoA	35.6 h (90% → 100%)	8.3 h (0%)	calculated from above[3]
Cdc42[2]	15 h (>90%)	11 h (67%)	(Backlund, 1997)
Cdc42	15 h (100% → 100%)	6.2 h (0%)	calculated from above[3]
Cdc42	16.9 h (90% → 100%)	5.0 h (0%)	calculated from above[3]

[1] Isoprenylcysteine carboxyl methyltransferase (Icmt) defective mouse cells expressing human Icmt and derived *Icmt* defective cells. Half-lives detected using "pan-Rho" and "pan-Ras" antibodies, respectively (Bergo et al., 2004). [2] Mouse cells. Chemical methylation inhibition through 3-deazaaristeromycin (DZAri) (Backlund, 1997). [3] Numerically calculated based on the above data. These results should be interpreted as rough indications. Calculation assumptions: 1) The reported "half-life" is the time until the mixture of methylated and unmethylated GTPase is reduced by 50%. 2) Methylated and unmethylated GTPase both decay by simple exponential decay, each with their own constant rate. 3) Reported methylation percentages of >90% are explored using 90% and 100% as bounds, as indicated.

2 Supplementary figures

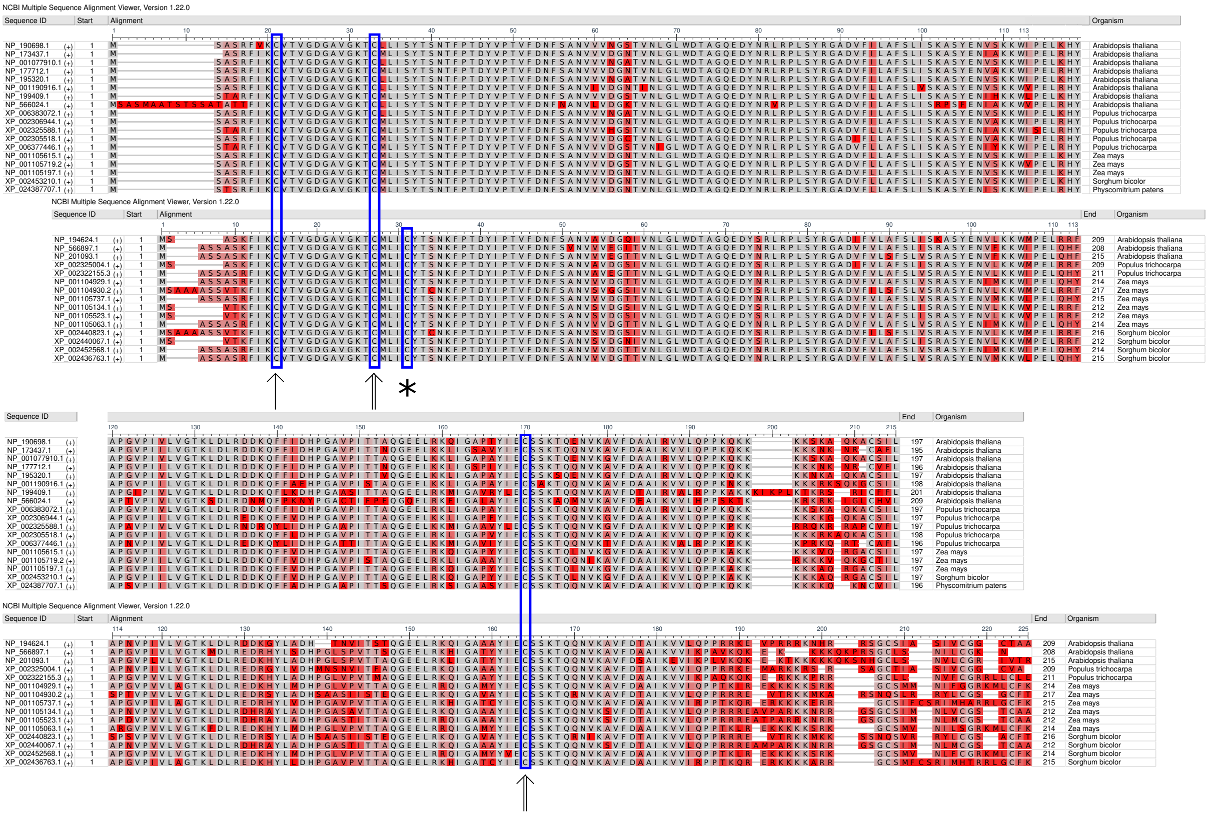

Figure S1: Multiple sequence alignment showing conserved Cys (C) residues in type-I (top blocks) and II (bottom blocks) ROPs. Conserved Cs that are shared between type-I and type-II (and animals) are indicated with arrows. Double arrows indicate sites of confirmed S-acylation coupled to activation status (Sorek et al., 2017, 2010). An additional C that is conserved within the type-II ROPs is indicated with *. Alignment made with NCBI COBALT. Shades of red indicate deviation from the majority amino acid (not considering amino acid properties). As a result, variable regions are overall coloured red, conserved regions are overall coloured grey.

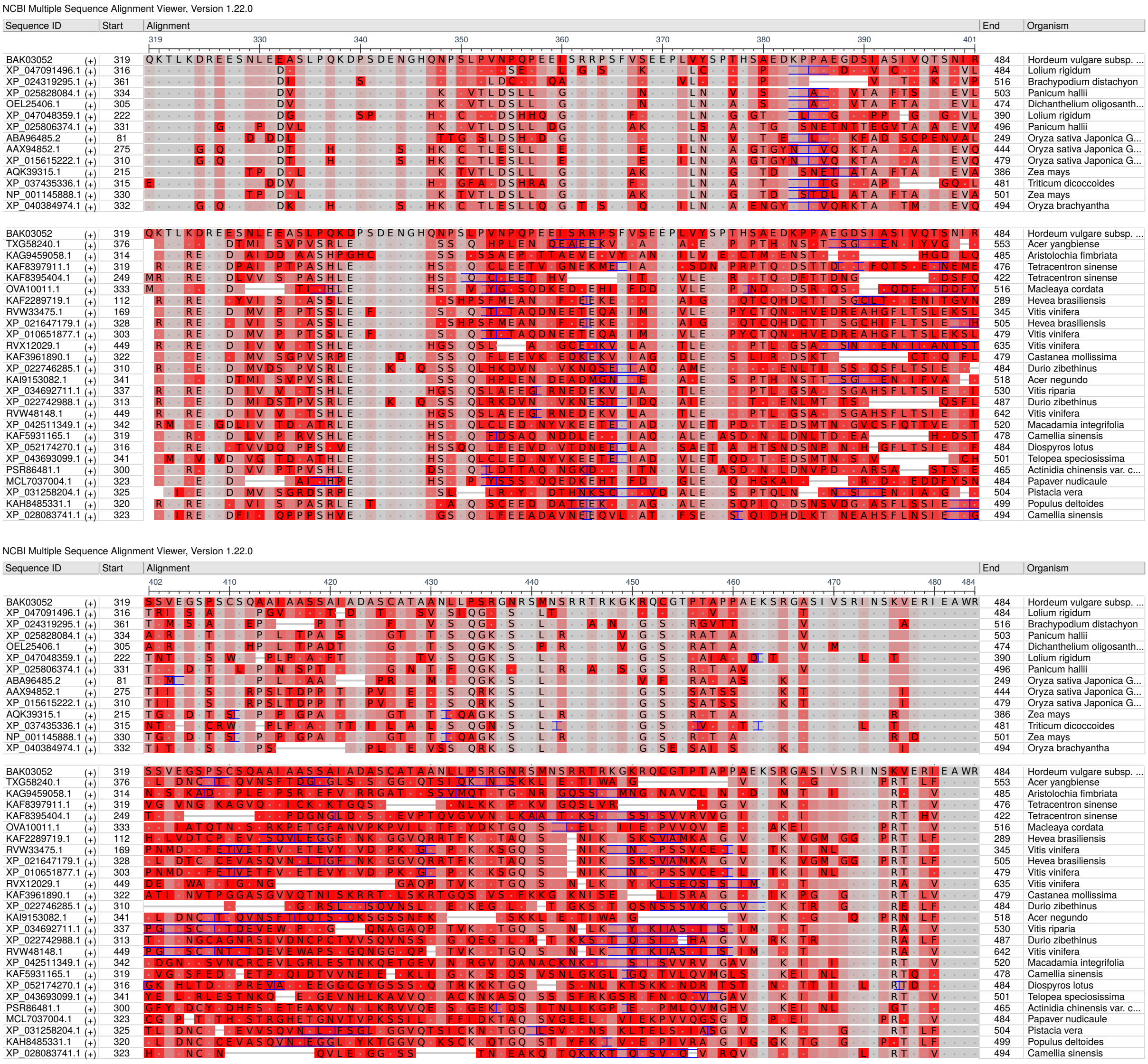

Figure S2: Multiple sequence alignment showing BLAST hits to the C-terminal domain (residues 319-484) of HvMAGAP1 (NCBI BLASTp to the clustered nr database, accessed December 17, 2022). Top blocks: all annotated hits with an E-value $\leq 10^{-45}$, which all are RhoGAPs from monocots. Bottom blocks: all hits outside the monocots (basal flowering plants and eudicots). These all had E-values between 10^{-15} and 10^{-8}. The top row in all blocks is HvMAGAP1. Colour scheme as in Supplementary Fig. S1: variable regions are overall coloured red, conserved regions are overall coloured grey. Dots indicate identical amino acids.

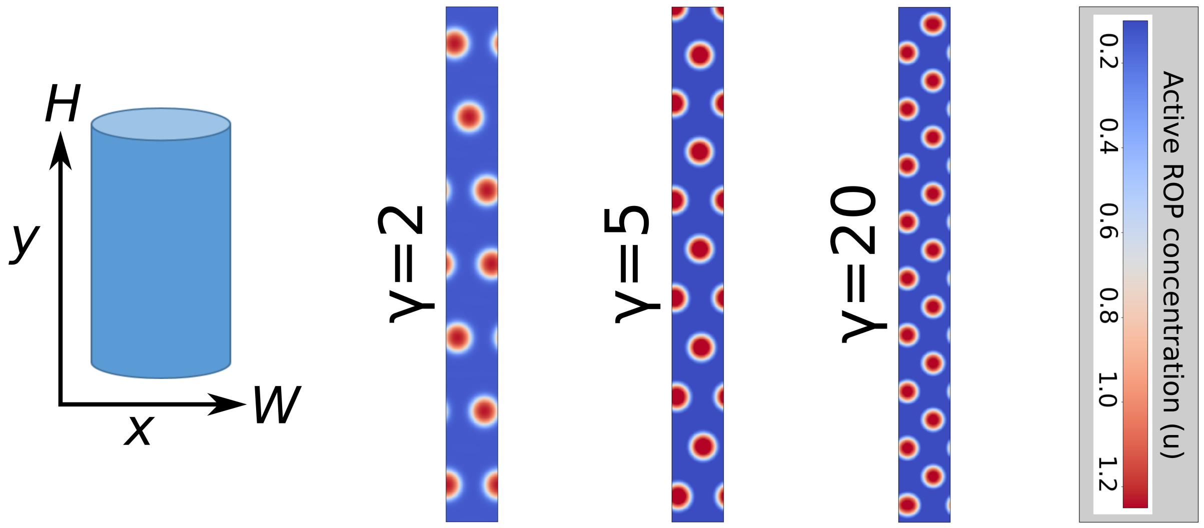

Figure S3: Steady state patterns for different values of feedback activation parameter γ, generated by the wave pinning model with turnover (WPT), a model that allows coexisting clusters. All parameters are as in Jacobs et al. (2020), Fig. 2 top left panel, except for the domain width, which has been reduced by a factor 6, and γ, which is indicated in the figure.

References

Backlund, P. S. (1997). Post-translational processing of RhoA: Carboxyl methylation of the carboxyl-terminal prenylcysteine increases the half-life of RhoA. *Journal of Biological Chemistry*, 272(52):33175–33180.

Bergo, M. O., Gavino, B. J., Hong, C., Beigneux, A. P., McMahon, M., Casey, P. J., and Young, S. G. (2004). Inactivation of Icmt inhibits transformation by oncogenic K-Ras and B-Raf. *The Journal of Clinical Investigation*, 113(4):539–550.

Bracha-Drori, K., Shichrur, K., Lubetzky, T. C., and Yalovsky, S. (2008). Functional analysis of arabidopsis postprenylation caax processing enzymes and their function in subcellular protein targeting. *Plant physiology*, 148(1):119–131.

Jacobs, B., Molenaar, J., and Deinum, E. E. (2020). Robust banded protoxylem pattern formation through microtubule-based directional rop diffusion restriction. *Journal of Theoretical Biology*, 502:110351.

Sorek, N., Poraty, L., Sternberg, H., Buriakovsky, E., Bar, E., Lewinsohn, E., and Yalovsky, S. (2017). Corrected and republished from: Activation status-coupled transient s-acylation determines membrane partitioning of a plant rho-related gtpase. *Molecular and cellular biology*, 37(23):e00333–17.

Sorek, N., Segev, O., Gutman, O., Bar, E., Richter, S., Poraty, L., Hirsch, J. A., Henis, Y. I., Lewinsohn, E., Jürgens, G., et al. (2010). An s-acylation switch of conserved g domain cysteines is required for polarity signaling by rop gtpases. *Current Biology*, 20(10):914–920.
